# Integrative Systems Biology Approaches to Identify Potential Biomarkers and Pathways of Cervical Cancer

**DOI:** 10.3390/jpm11050363

**Published:** 2021-04-30

**Authors:** Arafat Rahman Oany, Mamun Mia, Tahmina Pervin, Salem Ali Alyami, Mohammad Ali Moni

**Affiliations:** 1 Department of Biotechnology and Genetic Engineering, Faculty of Life Science, Mawlana Bhashani Science and Technology University, Tangail 1902, Bangladesh; arafatr@outlook.com (A.R.O.); mamunmiabge2010@gmail.com (M.M.); 2 Aristopharma Limited, Dhaka 1000, Bangladesh; 3 RT-PCR Laboratory, AICHI Hospital Ltd., Dhaka 1230, Bangladesh; 4 Biotechnology and Genetic Engineering Discipline, Life Science School, Khulna University, Khulna 9208, Bangladesh; tahminar@outlook.com; 5 Department of Mathematics and Statistics, Faculty of Science, Imam Mohammad Ibn Saud Islamic University (IMSIU), Riyadh 13318, Saudi Arabia; saalyami@imamu.edu.sa; 6 WHO Collaborating Centre on eHealth, UNSW Digital Health, School of Public Health and Community Medicine, Faculty of Medicine, University of New South Wales, Sydney, NSW 2052, Australia

**Keywords:** systems biology, cervical cancer, prognostic biomarker, differentially expressed genes

## Abstract

Nowadays, cervical cancer (CC) is treated as the leading cancer among women throughout the world. Despite effective vaccination and improved surgery and treatment, CC retains its fatality rate of about half of the infected population globally. The major screening biomarkers and therapeutic target identification have now become a global concern. In the present study, we have employed systems biology approaches to retrieve the potential biomarkers and pathways from transcriptomic profiling. Initially, we have identified 76 of each up-regulated and down-regulated gene from a total of 4643 differentially expressed genes. The up-regulatory genes mainly concentrate on immune-inflammatory responses, and the down-regulatory genes are on receptor binding and gamma-glutamyltransferase. The involved pathways associated with these genes were also assessed through pathway enrichment, and we mainly focused on different cancer pathways, immunoresponse, and cell cycle pathways. After the subsequent enrichment of these genes, we have identified 12 hub genes, which play a crucial role in CC and are verified by expression profile analysis. From our study, we have found that genes LILRB2 and CYBB play crucial roles in CC, as reported here for the first time. Furthermore, the survivability of the hub genes was also assessed, and among them, finally, CXCR4 has been identified as one of the most potential differentially expressed genes that might play a vital role in the survival of CC patients. Thus, CXCR4 could be used as a prognostic and/or diagnostic biomarker and a drug target for CC.

## 1. Introduction

Cervical cancer (CC), classified as the second most prominent cancer, is one of the most recurrently diagnosed cancers in terms of prevalence and sources of cancer-related deaths in women worldwide [1]. According to World Health Organization (WHO), cervical cancer represents almost 6.6% of all cancers of females, with an estimated 570,000 new cases in 2018 where low- and middle-income countries experienced an average of 90% deaths [2,3,4]. Developed countries have also experienced the fatality of this cancer; for instance, only the USA accounted for almost 13,170 newly diagnosed cervical cancer cases and 4250 deaths in 2019 [5].

Human papillomavirus (HPV) infection suggested as the first and foremost cause of cervical cancer pathogenesis [6,7,8], and studies reported that HPV is responsible for genome aberrations and increases specific target-wise gene expression [9]. Apart from mutations in P53, PIK3CA, PTEN genes, an altered number of oncogenes copies have been reported as the core factor of cervical carcinoma progression [10,11,12]. Other risk factors such as smoking, pregnancy history, and long-term use of oral contraceptives have also been conveyed [13].

The available treatment strategies such as surgery, radiotherapy, and chemotherapy are thought of as the prodigious hope for cervical cancer patients, however about 75% of patients develop further progression and/or recurrent tumors [14,15]. Disease heterogeneity of patients is another challenge to apply a specific treatment method [5].

Nevertheless, there are some available diagnostic and prognostic methods of cervical carcinoma specific to the time of onset, including HPV test, Pap test, and visual inspection with acetic acid (VIA), according to which cervical cancer may be suspected. Since several Pap smears are non-diagnostic or incorrectly negative throughout the case of invasive cancer, a histopathology examination might be obtained from any suspected lesion [16]. With cervical biopsies lacking an estimated 33–50% of high-grade cervical lesions, more sensitive and specific screening tests are needed, which may be possible in the form of screening biomarkers and tumor antigens [17]. There are several clear clinical signs in the early stage of some patients with cervical cancer, but they can be easily ignored by patients. Therefore, some patients lose better treatment opportunities. Accordingly, there is a requirement to elucidate the molecular mechanisms underlying cervical cancer development and progression, providing a basis for finding potential drug targets and diagnosing biomarkers of CC [18,19]. Therefore, there is a pressing need to identify new suitable molecular markers or models to predict the diagnosis/prognosis of cervical cancer [20].

A series of studies have already been conducted by relying on gene expression patterns to sort out auspicious molecular gene signatures to use as a recurrence prediction tool. A signature of 7-gene series was identified in the early stage, [21] and a predictive prognostic model for recurrent tumor had been constructed, which is composed of a 12-gene series [22]. Through the analysis of long non-coding RNA (lnc-RNA), it was revealed that nine-gene signature sets were used to predict patients’ chance to develop recurrent tumors [23]. However, searching for an effective series of gene sets is to be under investigation that can be used as a promising prognosis and/ diagnosis purpose to mitigate the disease outcome at the very early stage.

Nowadays, an integrative systems biology approach is a promising technique, which has been applied to predict novel molecular oncogenes and gene signatures using existing gene expression profiles from Gene Expression Omnibus (GEO) [24,25,26,27,28,29,30,31].

In the current research, we have employed systems biology approaches to explore the differentially expressed genes (DEGs), gene network, pathways, and protein–protein interactions unique to CC to retrieve potential biomarkers and pathways of cervical carcinoma.

## 2. Materials and Methods

**Figure 1:**
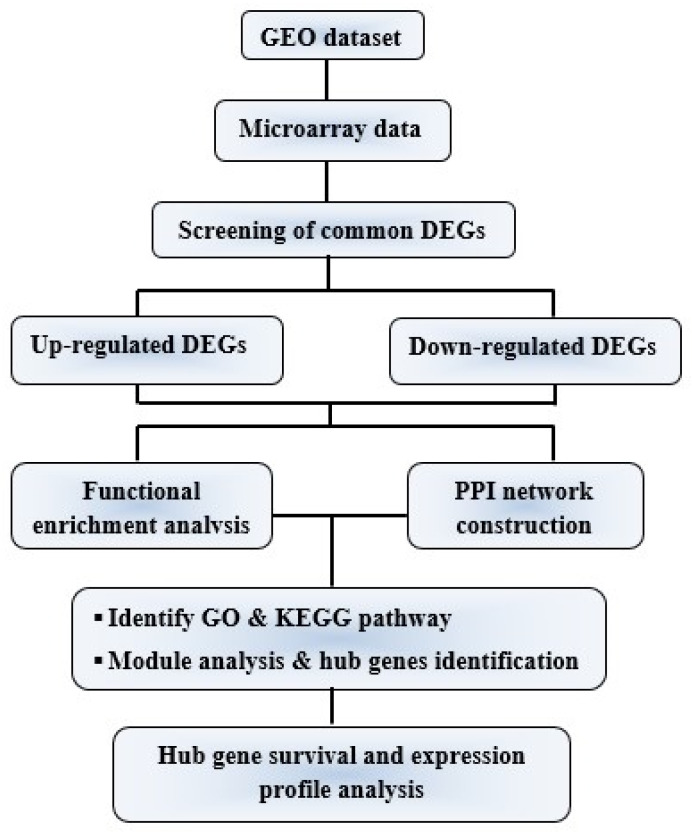
Flow diagram of integrative bioinformatics analytical approach applied in this study.

The entire procedure of the integrative systems biology analytical approach to identify novel molecular gene signatures and pathways of cervical cancer is shown in Figure 1 through the schematic diagram.

### 2.1. Data Retrieval

The gene expression profile (GSE148747) of primary fibroblasts from the normal region vs. tumorous region of the human uterine cervix, based on the platform of GPL4133 (Agilent-014850 Whole Human Genome Microarray 4x44K G4112F), were collected from the Gene Expression Omnibus (GEO) (https://www.ncbi.nlm.nih.gov/geo/ accessed on 17 April 2020) database, a public repository that provides free access to a full set of microarray, next-generation sequencing and other forms of high-throughput functional genome data submitted by the different research group [32]. A total of eight samples were used in this dataset (GSE148747). The GSM4478163, GSM4478166, GSM4478167 GSM4478168, and GSM4478170 were used as normal primary fibroblast, and the GSM4478164, GSM4478165, and GSM4478169 were used as tumor-associated fibroblast.

### 2.2. Screening of Differentially Expressed Genes (DEGs)

We utilized the GEO2R (http://www.ncbi.nlm.nih.gov/geo/geo2r/ accessed on 17 April 2020) statistical tool to calculate and assess the genes that were expressed differently between the primary fibroblasts from the normal region and tumorous region of the human uterine cervix [33]. The Benjamini and Hochberg (false discovery rate) and *t*-test methods were utilized with the GEO2R tool to calculate the FDR and *p*-values, respectively, to identify the DEGs [34]. We considered *p*-value *p* < 0.05 and a logFC (large-scale fold changes) > 1 to be statistically significant for the DEGs, and a logFC ≥ 1, logFC ≤ −1 were considered to indicate up-regulated and down-regulated DEGs, respectively [35,36,37]. The resultant DEG dataset was collected and used for further analysis. Including all of the DEGs identified in the samples, we constructed a volcano plot by using the pheatmap package in R language.

### 2.3. Functional Enrichment of Gene Sets

The initial ontology of gene (GO) and KEGG pathway enrichment analyses of the DEGs were annotated (*p* < 0.05) using the online bioinformatics tool DAVID v6.8 [38]. The human genome was selected as the background parameter, and Affymetrix based identification was selected as identifier. The KEGG pathway enrichment analyses of the DEGs were cross-checked using the NetworkAnalyst online tool [39]. Gene Ontology (GO) study is a frequently used approach for the functional studies of large-scale transcription or genomic data [40,41]. Similarly, the Kyoto encyclopedia of genes and genomes (KEGG) is generally used to understand metabolic pathways for gene annotation [42,43].

### 2.4. PPI Network Construction

The online database STRING (v11.0, http://www.string-db.org/ accessed on 17 April 2020) was used to construct the PPI network of the proteins encoded by DEGs. The String is an online repository with 24,584,628 proteins from 5090 organisms to predict the relationship between genes [44]. The combined score was set at less than 0.75 (medium confidence score) to be considered significant.

### 2.5. Selection of Central Hub Proteins from the PPI Network

The obtained PPI networks were visualized by Cytoscape (http://www.cytoscape.org/ accessed on 17 April 2020) [45]. The Cytoscape plug-in Molecular Complex Detection (MCODE) [46] was applied to obtain significant modules with an established score of greater than 3 and nodes of greater than 4. In the PPI network, the number of edges involved determines the degree value of the nodes; nodes with high degree values were considered to be hub genes. We mapped the hub genes to evaluate their PPI information. We use cytoHubba [47] (a Cytoscape plugin) to evaluate hub genes from the constructed PPI network. The cytoHubba is a tool that uses 11 specific methods to calculate hub genes from the PPI network; in this study, we use degree score to identify hub genes.

### 2.6. Hub Gene Survival and Expression Profile Analysis

A comprehensive online platform called Gene Expression Profiling Interactive Analysis (GEPIA2) [48] provides fast and customized delivery of functionalities based on TCGA (The Cancer Genome Atlas) and genotype-tissue expression (GTEx) data. GEPIA2 evaluates the survival effect and the expression profile analysis of differentially expressed genes in a given cancer sample. The overall survival effect of hub genes in CC was estimated by calculating the log-rank *p*-value and the HR (hazard ratio—95% confidence interval) using GEPIA2 single-gene analysis. On the other hand, the relative expression levels of the hub genes were selected based on the Log2FC cutoff value of <1 and the q-value Cutoff was <0.01.

## 3. Results and Analysis

### 3.1. DEG Identification

**Figure 2:**
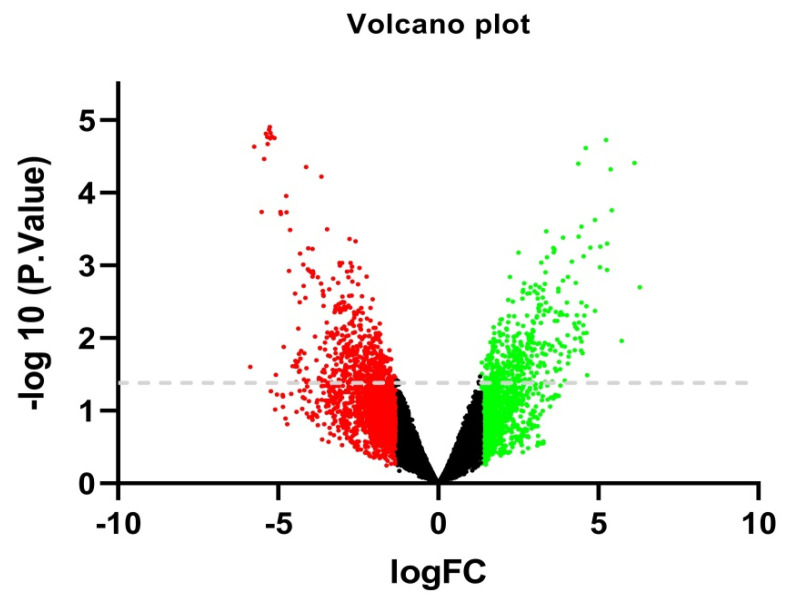
Volcano plot of the DEGs from the GSE148747 dataset. X-axis: logFC, large-scale fold changes; y-axis: -log10 of the *p*-value is showing the statistical significance. The green points represent the up-regulated genes while red points are for the down-regulated genes.

The expression profiling was performed on the CC gene dataset GSE148747, which was retrieved from GEO and the overall patients’ information is shown in Table 1. To identify the DEGs from these two groups, we conducted a GEO2R web-server to calculate the *p*-values and log2FC values. The resulting genes that met the cutoff criteria (logFC ≥ 1, logFC ≤ −1, and *p*-value *p* < 0.05) were considered as DEGs. Overall, 4643 genes were identified from the GEO dataset using the GEO2R tool. The volcano plot was constructed by comparing the two groups and is depicted in Figure 2. Finally, among them, a total of 152 DEGs were identified based on the cutoff criteria (logFC ≥ 1, logFC ≤ −1 and an adjusted *p*-value *p* < 0.05), where 76 and 76 were up-regulated and down-regulated, respectively (Tables S1 and S2).

### 3.2. Functional Analysis of DEGs

**Figure 3:**
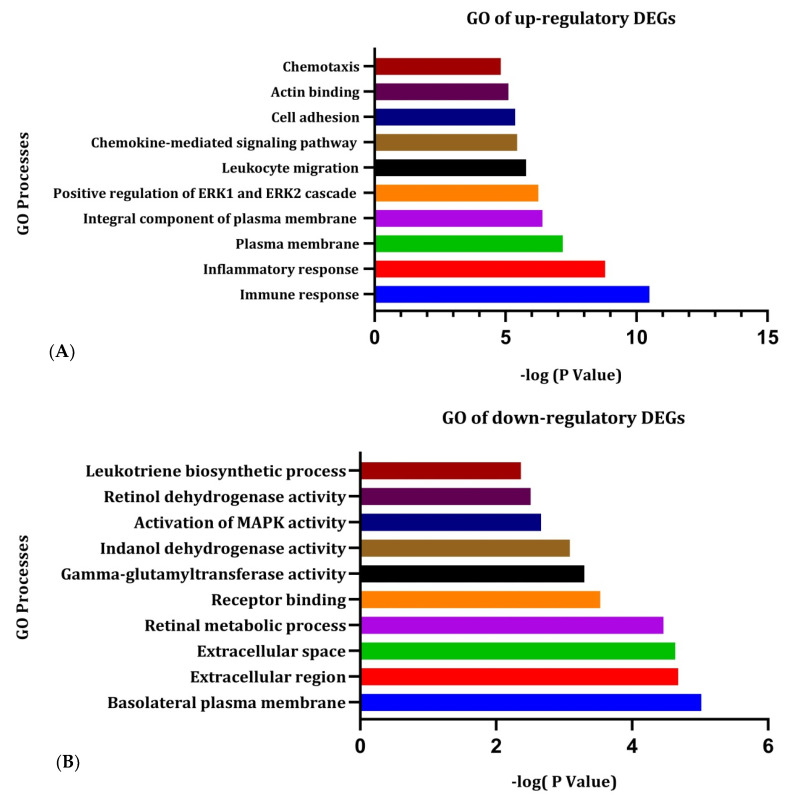
Functional analyses of the differentially expressed genes (DEGs) in cervical cancer. In figure (**A**), GO of up-regulatory DEGs and in (**B**). GO of down-regulatory DEGs. Each of the GO processes was plotted based on the -log (*p*) value.

**Figure 4:**
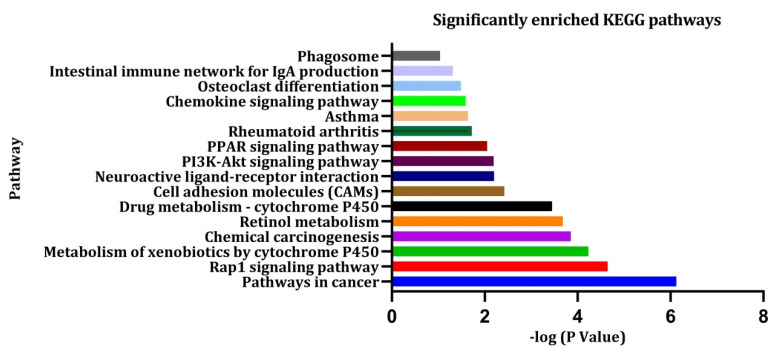
The enriched KEGG pathways generated from the DEGs.

By using the DAVID database, the top 10 enrichment analysis outcomes were screened for the up-regulated and down-regulated DEGs of the GO analysis. The biological process (BP), cellular component (CC), and molecular function (MF) enrichment analysis outcomes are displayed in Figure 3A,B. DAVID and NetworkAnalyst analysis of KEGG pathway outcomes for the significantly enriched DEGs are depicted in Figure 4. The overlapped gene lists of up- and down-regulated genes that were involved in GO are shown in supplementary Tables S3 and S4, and the gene-lists of the KEGG pathway are shown in supplementary Table S5, respectively.

### 3.3. PPI Network Construction

**Figure 5:**
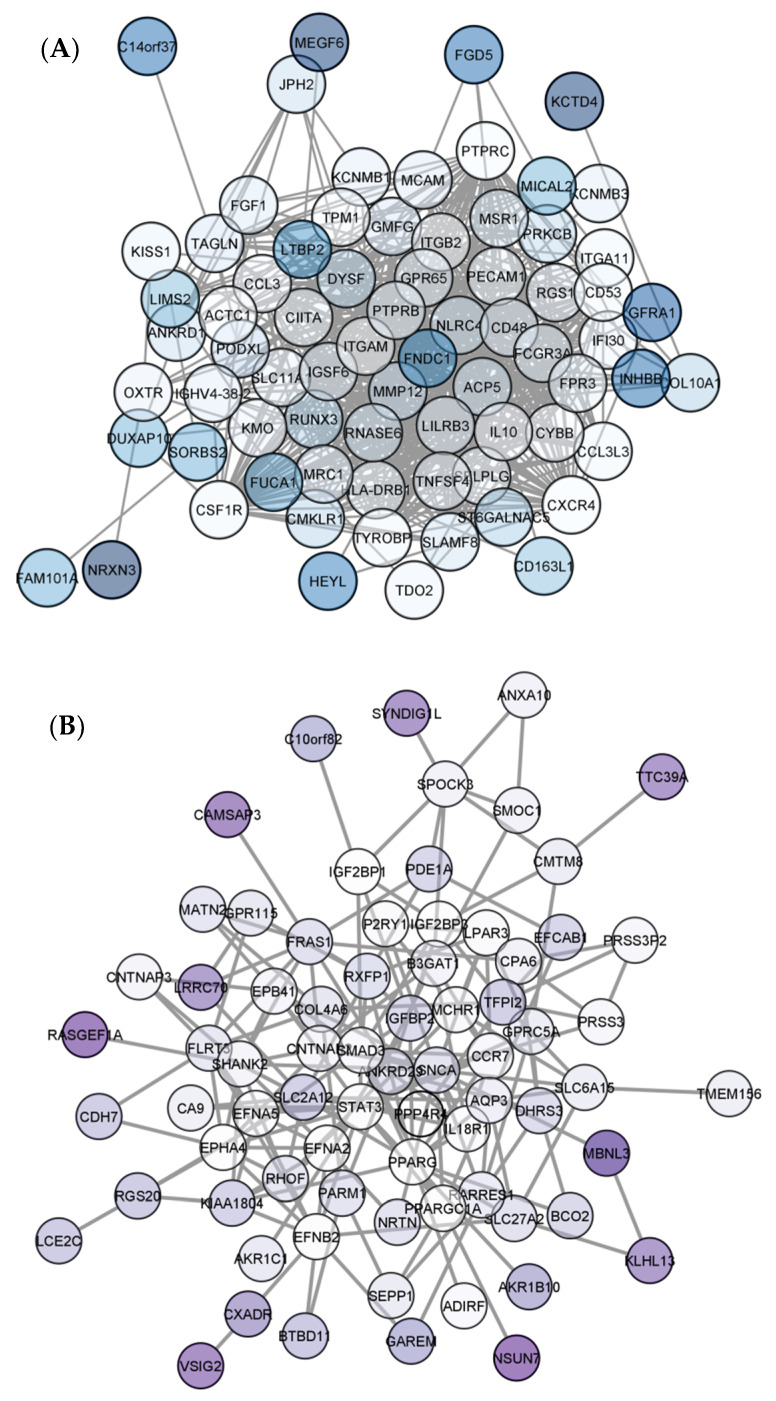
Visualization of the protein–protein interaction network of CC. In figure (**A**), the PPI network for up-regulated genes with 76 nodes and 656 edges and in figure (**B**) the PPI network for down-regulated genes with 76 nodes and 151 edges.

To evaluate the PPIs between the DEGs, we used the STRING tool to identify the PPI networks for both the up- and down-regulated genes. Thereafter, the resulting PPI network from STRING was exported as a “.txt” file and imported as a.csv file into Cytoscape v3.8.0 software for visualization. After subsequent enrichment of each of the networks, we have generated a merged network of PPI consisting of 152 nodes and 1206 edges (Figure S1). The PPI network for up-regulated DEGs had 76 nodes with 656 edges (Figure 5A) and the PPI network for down-regulated DEGs had 76 nodes with 151 edges between the nodes (Figure 5B).

### 3.4. Modules and Hub Proteins Identification

**Figure 6:**
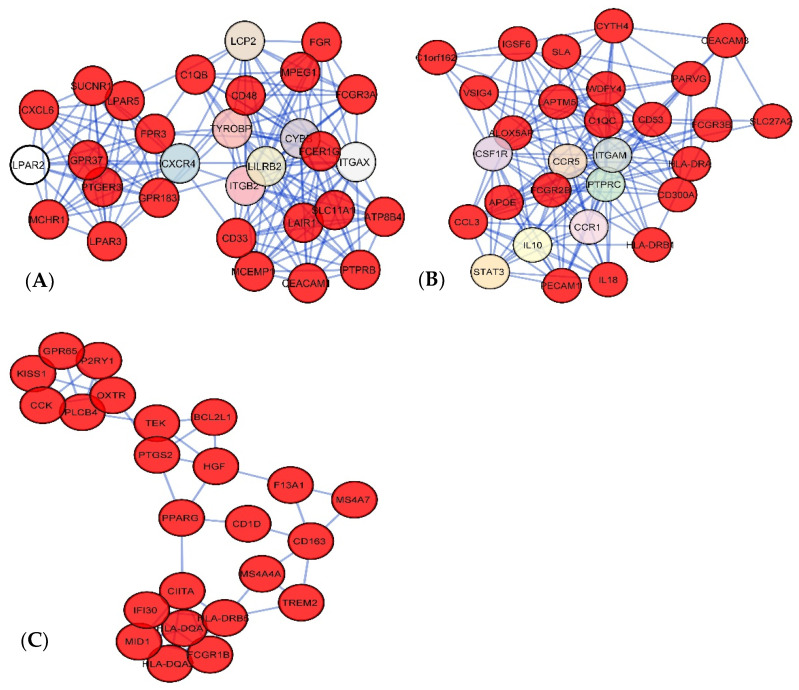
Module analysis of PPI network. (**A**) Module 1 had 30 nodes with 187 edges. (**B**) Module 2 had 29 nodes and 166 edges. (**C**) Module 3 had 24 nodes and having 57 edges.

**Figure 7:**
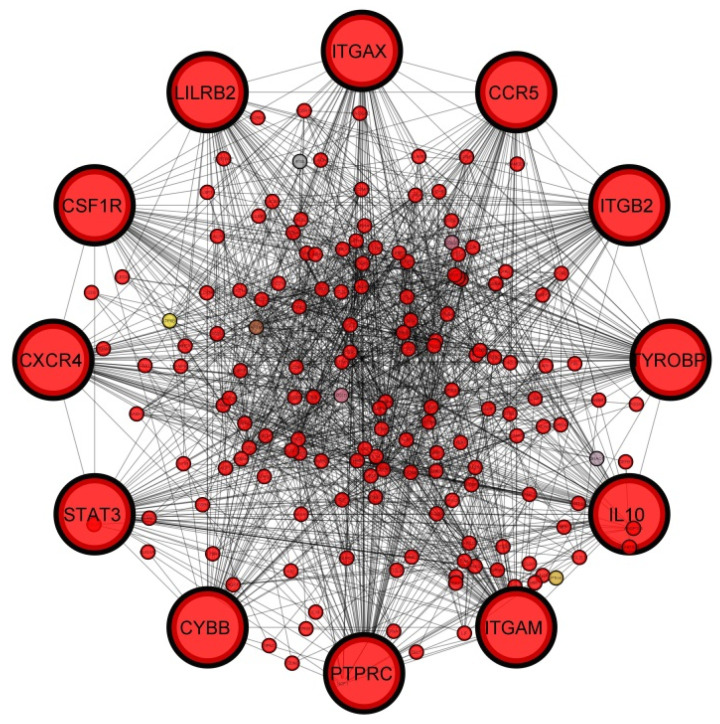
PPI network with identifying hub genes. The highlighted 12 nodes represent the hub genes. The degree score method of cytoHubba considers 94 nodes in this network from the merged PPI network.

We identified three significant modules from the merged PPI network by using the Cytoscape plugin MCODE. Module 1 had 30 nodes with 187 edges (Figure 6A); on the other hand, Module 2 and 3 had 29 and 24 nodes with 166 and 57 edges, respectively (Figure 6B,C). The DEGs of the modules had an important role to enrich significant GO terms ‘protein binding’ and ‘cytoplasm’. We also identified the top 12 hub genes (Table 2) from the PPI network using the cytoHubba tool and are depicted in Figure 7. The topological parameters of the twelve molecular hub genes of the PPI network are also shown in Table 2, including the clustering coefficient, degree, the betweenness centrality, and the closeness centrality for the individual gene.

### 3.5. Survival and Expression Level of the Hub Genes

**Figure 8:**
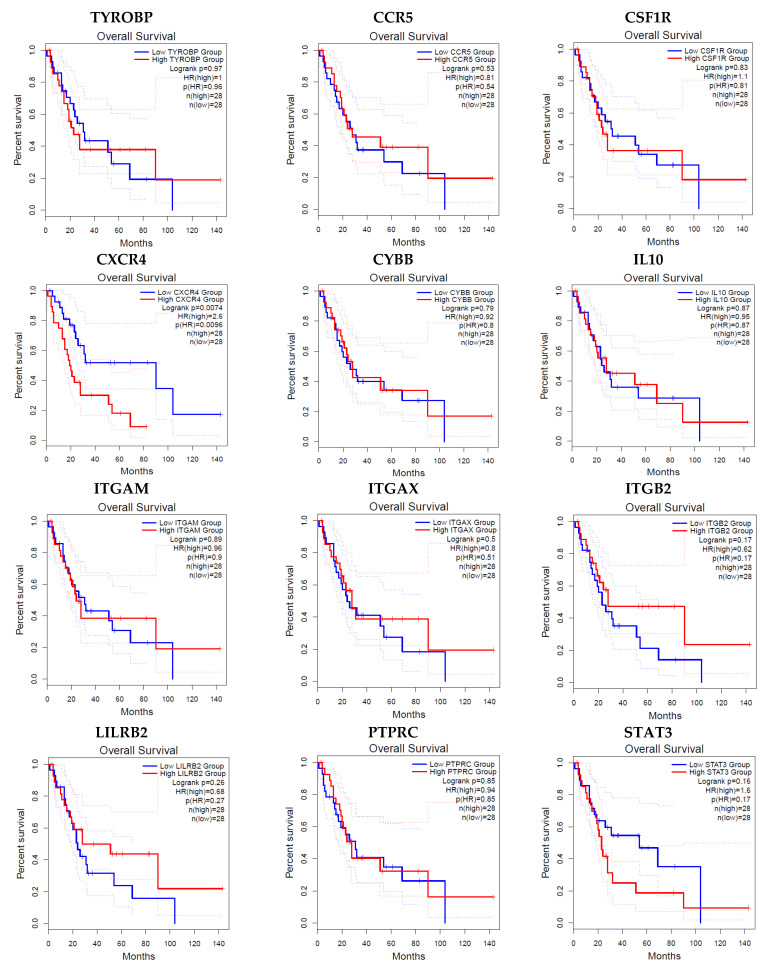
Kaplan–Meier overall survival analysis of the hub genes expressed in CC. Curves were generated using Gene Expression Profiling Interactive Analysis based on The Cancer Genome Atlas database (*p* ≤ 0.01).

**Figure 9:**
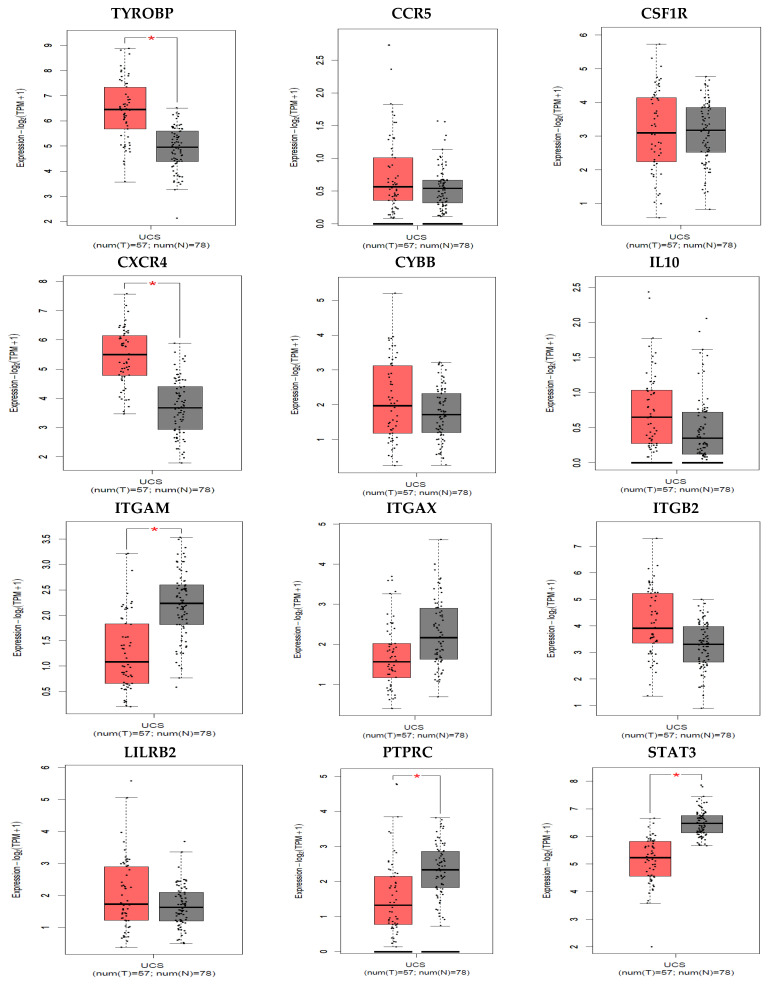
Relative expression levels of hub genes expressed in CC. Red and black boxes represent the relative expression levels of genes in the tumor and normal samples, respectively. The y-axis represents the relative expression levels of genes in terms of log2 (TPM + 1) (tumor samples, 57; normal samples, 78 from GEPIA; *p* ≤ 0.01). TPM, transcripts per million.

GEPIA survival assessment was used to inspect the overall association with survival of 12 hub genes from both the up- and down-regulated DEGs and is depicted in Figure 8. Among all the twelve genes, only CXCR4 showed lower overall survival in the higher expression group and had the Hazard Ratio (HR) of 2.6. On the other hand, from the expression level of the hub genes we have identified that the four hub genes ITGAM, ITGAX, PTPRC and STAT3 were up-regulated in the normal cells and the other eight hub genes were up-regulated in the tumor cells (Figure 9).

## 4. Discussion

The detection of early-stage CC has an admirable prognosis compared to advanced-stage. The early-stage cases were cured significantly through surgery, chemo-radiation, or a blend of treatment approaches where the advanced level CC patients suffered most because of the frequent recurrences and incurable nature of CC [1].

The patterns of the DEGs, from both tumor and control, were visualized by the volcano plot (Figure 2). The green points represent the up-regulated genes, and the red points are the down-regulated genes.

After subsequent enrichment of all the DEGs, we have found some significant up-regulatory GOs such as immune response, inflammatory response, chemokine-mediated signaling pathway, positive regulation of ERK1 and ERK2 cascade, and chemotaxis in the biological process. For the cellular component, the plasma membrane and integral module of the plasma membrane are enriched significantly. The major molecular function up-regulated in the DEGs were immune response and inflammatory response.

On the other hand, down-regulatory GOs such as retinal metabolic process, activation of MAPK activity, and leukotriene biosynthetic process were found in the biological process. The major molecular function down-regulated in the DEGs were receptor binding, gamma-glutamyltransferase activity, and indanol dehydrogenase activity.

From the pathway analysis, we have found that most of the pathways were mainly concentrated in different cancer pathways, immunoresponse, and cell cycle pathways (Figure 4 and Table S5).

We identified 12 hub genes based on degree value, clustering coefficient, betweenness centrality, and closeness centrality from the merged PPI analysis; these 12 genes PTPRC, ITGAM, IL10, TYROBP, ITGB2, CCR5, ITGAX, CSF1R, LILRB2, CXCR4, STAT3, and CYBB had the diagnostic value which distinguishes CC from normal individuals (Table 2). A higher percentage of cervical tumors occupied by PTPRC+ cells were strongly associated with enhanced tumor-infiltration by Tbet+ cells and Foxp3+ cells. Longer disease-free and disease-specific survival were reported in the type I-oriented PTPRC+ cell to infiltrate occupied tumor areas [49]. ITGAM, ITGB2 genes play a pivotal role in cell adhesion in multiple myeloma and are reported as potential diagnostic markers [50]. Type-2 cytokine, IL-10 shows immunosuppressive functions and is capable of stimulating tumor growth, and cervical tumor biopsies showed increased presence for mRNA for IL-10 [51]. The chemotactic receptors, CCR5 expressed in CD8+ T cells which preferentially proliferated in cervical cancer [52], and the proliferation and invasion of cervical cancer can be arrested by down-regulating the expression of CCR5 [53]. Similarly, the reduced level of CSF1R protein significantly contributes to suppressing cervical cancer cell proliferation and motility, and induces apoptotic cell death [53]. Therefore, CCR5 and CSF1R might be the new targets for cervical cancer treatment.

STAT3 accumulation was observed in specific sites, especially in basal and suprabasal layers of HPV16-positive early pre-cancer lesions, and STAT3 expression and activity were distinctively higher in poorly differentiated lesions [54]. Thus, it suggests that the STAT3 gene might be used as a prognostic marker in cervical cancer prognosis at a very primary stage.

On the other hand, the two genes—LILRB2 and CYBB—have not been previously reported to be associated with cervical cancer. These genes need to be independently validated before they can be useful for evaluating the prognosis and/or diagnosis of CC patients.

A cervical tumor invades in adjacent tissues and subsequently into distant organs initiated by the expression of CXCR4 through the link of another factor CXCL12 [55]. Additionally, CXCR4-deficient cells had lower expression of the proliferation marker Ki-67 and decreased ability to engraft into lungs and spleen [56,57].

The CXCR4 down-regulation and silencing impairs cell adhesion, preventing metastasis. MMP-9 levels are substantially lower in CXCR4-deficient cells, and CXCR4 down-regulation reduces tumor development in vivo [56]. The epigenetically silenced CXCR4 gene prevents cells from responding to the paracrine source of SDF-1, resulting in cell adhesion failure, which is one of the primary events in cervical carcinogenesis metastasis and development [57,58]. CXCR4 receptor silencing by RNA interference, on the other hand, resulted in a reduction in the cells’ ability to signal, cause migration, and form holoclone-like colonies while having little effect on their viability or multiplication [56]. Smith et al. have reported that the initiation of cell proliferation and/or survival of cervical cancer cells is not possible without the CXCR4 receptor [59]. From the Kaplan–Meier overall survival analysis, the level of only CXCR4 expression was significantly related to the overall survival of patients with cervical cancer, while the rest of the hub genes’ survivability was lower in the high expression group (Figure 8). According to our analysis from DEGs, among the identified key 12 hub genes, only STAT3 showed the down-regulatory expression patterns whilst all the remaining genes were up-regulated. For the validation of this analysis, the GEPIA expression level of the hub genes were also employed and gene- ITGAM, ITGAX, PTPRC along with STAT3 were found in lower expression levels in tumor cells (Figure 9).

With the development of integrative systems biology approaches, researchers are evaluating the significance of gene biomarkers to improve the worsening condition of cancer diseases, e.g., cervical cancer. The microarray dataset of mRNA and miRNA analysis revealed RhoB and STMN1 genes as potential targets for cervical cancer diagnosis and treatment [60]. Recently, X Li et al. suggested a distinct pathway related to DNA replication and MCM5 as an independent prognostic gene biomarker [20]. An analysis was conducted to build a PPI network between down-regulated and up-regulated genes to reveal the interactions and functional activities of them where the top 5 genes—ITGAM, PTPRC, ITGAX, TYROBP, and C3AR1—were reported as the most important genes. However, only PTPRC showed overall survival in cervical cancer [61]. To our best knowledge, this is the first study ever conducted with the abovementioned microarray datasets (GSE148747) to identify the potential gene biomarker in cervical cancer.

Finally, from the overall analysis and discussion we proposed that CXCR4 could be a novel target for the prevention of cervical carcinoma growth and metastasis.

## 5. Conclusions

To summarize, our analysis from the current study provides evidence that candidate genes such as PTPRC, CXCR4, CCR5, and CSF1R and their enriched pathways, like the plasma membrane, inflammatory response, and chemokine-mediated signaling pathways might be involved in the pathogenesis of CC. Additionally, the genes LILRB2 and CYBB could be novel targets for CC prognosis or diagnosis. Moreover, from our analysis, we emphasized that CXCR4 might play a significant role in the metastasis of CC. Finally, we believe that the results obtained above can provide theoretical guidelines for future works in the laboratory. Further wet lab investigation is required to validate our findings to be used as a prognostic, diagnostic, and unique target to support the treatment management of CC.

## Figures and Tables

**Table 1:** Patients’ information in GSE148747 derived from the GEO database.

Group	Accession	Organism	Disease State	Cell Type
Normal	GSM4478163	Homo sapiens	Normal	Normal fibroblast
	GSM4478166	Homo sapiens	Normal	Normal fibroblast
	GSM4478167	Homo sapiens	Normal	Normal fibroblast
	GSM4478168	Homo sapiens	Normal	Normal fibroblast
	GSM4478170	Homo sapiens	Normal	Normal fibroblast
Tumor	GSM4478164	Homo sapiens	Cervical Cancer	Tumor-associated cervix fibroblasts
	GSM4478165	Homo sapiens	Cervical Cancer	Tumor-associated cervix fibroblasts
	GSM4478169	Homo sapiens	Cervical Cancer	Tumor-associated cervix fibroblasts

**Table 2:** Topological parameters for the twelve potential hub genes of the PPI network.

Gene Signature Name	Degree	Betweenness Centrality	Clustering Coefficient	Closeness Centrality	Stress
PTPRC	80	18,133.72223	0.23196	244.0667	167,664
ITGAM	79	14,447.28068	0.23337	242.4833	140,924
IL10	70	15,227.82113	0.18841	234.9833	145,074
TYROBP	69	6407.99793	0.29113	227.9833	78,988
ITGB2	66	9280.28036	0.28858	226.5595	95,454
CCR5	61	8309.33294	0.29836	225.8	98,026
ITGAX	60	4722.34853	0.29492	222.35	59,082
CSF1R	55	7708.90149	0.32727	221.5333	87,030
LILRB2	55	5622.88217	0.34007	217.1333	57,172
CXCR4	55	10,433.80294	0.24108	225.8333	95,538
STAT3	53	15,091.4144	0.20682	225.2833	126,744
CYBB	50	4529.93215	0.37469	218.95	54,562

## Data Availability

The datasets used in this article are publicly available as described in Section 2 (Materials and Methods).

## Supplementary Materials

The following are available online at https://www.mdpi.com/article/10.3390/jpm11050363/s1, Figure S1: All differentially expressed genes in PPI network as a merged illustration. This PPI network had 1206 edges for 151 nodes., Table S1: List of up regulated gene from GSE148747, Table S2: List of down regulated gene from GSE148747, Table S3: The functional analyses of up-regulated genes to identify the top 10 GO terms, Table S4: The functional analyses of down-regulated genes to identify the top 10 GO terms, Table S5: The gene lists of the significantly enriched KEGG pathways.
