# Supplementary material for: Integrative Systems Biology Approaches to Identify Potential Biomarkers and Pathways of Cervical Cancer
Source: J Pers Med. 2021 Apr 30;11(5):363. doi: 10.3390/jpm11050363 (PMC8147030; doi:10.3390/jpm11050363)
Supplement: Supplementary file 1 [file jpm-11-00363-s001.zip › Table S1, S2.pdf]

Table S1: List of up regulated gene from GSE148747

Serial No	ID	P Value	logFC	Gene Symbol	Gene Title
1	18444	0.0020007	6.29	FAM101A	Family with sequence similarity 101
2	13893	0.0001754	5.42	SLAMF8	SLAM family member 8
3	44509	0.0000478	5.38	IGH	Immunoglobulin heavy locus
4	14242	0.0011613	5.27	ANKRD1	ankyrin repeat domain 1 (cardiac muscle)
5	35069	0.0000189	5.24	OXTR	Oxytocin receptor
6	41953	0.0005541	5.06	KCNMB1	Potassium large conductance calcium-activated channel
7	15601	0.001067	5.05	KCNMB2	Potassium large conductance calcium-activated channel
8	35690	0.0042603	4.89	CYBB	Cytochrome b-245
9	27328	0.0005716	4.75	TNFSF4	Tumor necrosis factor (ligand) superfamily
10	26117	0.0036831	4.63	ACTC1	Actin
11	39609	0.0084978	4.63	ITGA11	Integrin
12	12708	0.0000245	4.61	CMKLR1	Chemokine-like receptor 1
13	43893	0.0085894	4.54	MMP12	Matrix metallopeptidase 12 (macrophage elastase)
14	32537	0.0067105	4.53	FNDC1	Fibronectin type III domain containing 1
15	44738	0.0002926	4.47	DUXAP10	double homeobox A pseudogene 10
16	35064	0.003277	4.47	FPR3	Formyl peptide receptor 3
17	9543	0.0061477	4.39	CCL3	Chemokine (C-C motif) ligand 3
18	4054	0.0004057	4.38	CSF1R	Colony stimulating factor 1 receptor
19	39647	0.0075405	4.33	FCGR3A	Fc fragment of IgG
20	7896	0.0017488	4.29	COL10A1	Collagen
21	32110	0.007047	4.28	ITGB2	Integrin
22	8902	0.0008923	4.17	CD48	CD48 molecule
23	14493	0.0014431	4.05	JPH2	Junctophilin 2
24	12088	0.0092106	4.04	CCL3L3	Chemokine (C-C motif) ligand 3-like 3
25	22873	0.0032642	4.01	FAM19A5	Family with sequence similarity 19 (chemokine (C-C motif)-like)
26	20694	0.0048894	4.01	TYROBP	TYRO protein tyrosine kinase binding protein
27	21491	0.0004167	3.9	PECAM1	Platelet/endothelial cell adhesion molecule
28	28644	0.001629	3.9	MRC1L1	Mannose receptor
29	28406	0.0047901	3.89	CD53	CD53 molecule
30	2842	0.00484	3.8	GFRA1	GDNF family receptor alpha 1
31	20108	0.002098	3.75	KISS1	Kiss-1 metastasis-suppressor
32	9080	0.0022977	3.75	CIITA	Class II
33	29706	0.0058166	3.71	IGSF6	Immunoglobulin superfamily
34	16143	0.00439	3.67	TAGLN	Transgelin
35	15184	0.0058569	3.63	NRXN3	Neurexin 3

Serial No	ID	P Value	logFC	Gene Symbol	Gene title
36	28629	0.0039083	3.51	RNASE6	Ribonuclease
37	14697	0.0042867	3.44	PTPRB	Protein tyrosine phosphatase
38	35795	0.0003417	3.37	SLC11A1	Solute carrier family 11 (proton-coupled divalent metal ion transporters)
39	40076	0.0032488	3.36	SORBS2	Sorbin and SH3 domain containing 2
40	4780	0.0018078	3.33	HEYL	Hairy/enhancer-of-split related with YRPW motif-like
41	1166	0.0041581	3.33	ITGAM	Integrin
42	3965	0.0048279	3.3	PODXL	Podocalyxin-like
43	11761	0.0021742	3.27	GMFG	Glia maturation factor
44	31894	0.0027773	3.27	TDO2	Tryptophan 2
45	33297	0.0009155	3.22	PRKCB	Protein kinase C
46	7963	0.0062027	3.21	LILRB3	Leukocyte immunoglobulin-like receptor
47	3676	0.0020057	3.16	CD163L1	CD163 molecule-like 1
48	19782	0.0042862	3.15	PTPRC	Protein tyrosine phosphatase
49	4666	0.0028637	3.11	DYSF	Dysferlin
50	22163	0.0024492	3.1	KMO	Kynurenine 3-monooxygenase (kynurenine 3-hydroxylase)
51	36966	0.0018708	3.04	RUNX3	Runt-related transcription factor 3
52	21523	0.0022891	3	ST6GALNAC5	ST6 (alpha-N-acetyl-neuraminyl-2
53	38894	0.0080595	2.97	CXCR4	Chemokine (C-X-C motif) receptor 4
54	27805	0.005914	2.95	MSR1	Macrophage scavenger receptor 1
55	10251	0.0088254	2.91	HLA-DRB1	Major histocompatibility complex
56	31350	0.0020135	2.88	INHBB	Inhibin
57	9432	0.0024402	2.85	MEGF6	Multiple EGF-like-domains 6
58	44609	0.0024661	2.78	LIMS2	LIM and senescent cell antigen-like domains 2
59	31687	0.0086988	2.76	RGS1	Regulator of G-protein signaling 1
60	33972	0.0017664	2.68	GPR65	G protein-coupled receptor 65
61	14933	0.0068428	2.65	FUCA1	Fucosidase
62	2855	0.0023223	2.64	KCTD4	Potassium channel tetramerisation domain containing 4
63	10450	0.0028615	2.62	ACP5	Acid phosphatase 5
64	22568	0.0086905	2.5	FGD5	Fyve
65	14332	0.0093114	2.45	TPM1	Tropomyosin 1 (alpha)
66	8646	0.0050184	2.37	MCAM	Melanoma cell adhesion molecule
67	38220	0.0098226	2.37	C14orf37	Chromosome 14 open reading frame 37
68	40243	0.0096059	2.36	NLRC4	NLR family
69	17528	0.0031616	2.32	ZCCHC5	Zinc finger
70	28936	0.004951	2.21	MICAL2	Microtubule associated monoxygenase
71	30297	0.003001	2.18	IFI30	Interferon
72	23594	0.0094464	2.16	SELPLG	Selectin P ligand

Serial No	ID	P Value	logFC	Gene Symbol	Gene title
73	28694	0.0088822	2.03	LTBP2	Latent transforming growth factor beta binding protein 2
74	14414	0.0064157	1.91	SPATA8	Spermatogenesis associated 8
75	9600	0.008327	1.9	IL10	Interleukin 10
76	11972	0.0086432	1.75	FGF1	Fibroblast growth factor 1 (acidic)

Table S2: List of down regulated gene from GSE148747

Serial No.	ID	P Value	logFC	Gene Symbol	Gene title
	10095	0.0184733	-1.638	GCHFR	GTP cyclohydrolase I feedback regulator
1	42781	0.0202829	-1.644	STAT3	Signal transducer and activator of transcription 3 (acute-phase response factor)
2	12129	0.0085633	-1.86	Synleurin	Synleurin
3	30384	0.0095157	-1.94	RASGEF1A	Rasgef domain family
4	23502	0.0086101	-1.95	SMAD3	SMAD family member 3
5	39392	0.0093845	-2.14	IGF2BP3	Insulin-like growth factor 2 mrna binding protein 3
6	35689	0.0079138	-2.17	SLC27A2	Solute carrier family 27 (fatty acid transporter)
7	17556	0.0094634	-2.17	RHOF	Ras homolog gene family
8	11828	0.0053909	-2.23	RGS20	Regulator of G-protein signaling 20
9	12826	0.0093265	-2.3	C9orf68	Chromosome 9 open reading frame 68
10	41571	0.0045149	-2.36	DKFZP564O0823	DKFZP564O0823 protein
11	16541	0.0026805	-2.38	KIAA1804	Mixed lineage kinase 4
12	18900	0.0074609	-2.42	PRSS3	Protease
13	13620	0.0076314	-2.42	TMEM90A	Transmembrane protein 90A
14	14853	0.0010926	-2.46	MCHR1	Melanin-concentrating hormone receptor 1
15	35755	0.0095534	-2.46	CNTNAP2	Contactin associated protein-like 2
16	27711	0.0067558	-2.48	LCE2C	Late cornified envelope 2C
17	42591	0.0018274	-2.51	IGFBP2	Insulin-like growth factor binding protein

					2
**Serial No.**	**ID**	**P Value**	**logFC**	**Gene Symbol**	**Gene title**
18	11008	0.0004676	-2.59	EPB41	Erythrocyte membrane protein band 4.1 (elliptocytosis 1
19	22840	0.0083849	-2.6	FRAS1	Fraser syndrome 1
20	15542	0.004764	-2.61	PRSS2	Protease
21	32181	0.0068091	-2.64	CMTM8	CKLF-like MARVEL transmembrane domain containing 8
22	8235	0.0085464	-2.66	PDE1A	Phosphodiesterase 1A
23	37540	0.0064638	-2.68	NSUN7	NOL1/NOP2/Sun domain family
24	27788	0.0016792	-2.69	SPOCK3	Sparc/osteonectin
25	13294	0.0058729	-2.7	NLRP7	NLR family
26	5618	0.0011989	-2.72	EFCAB1	EF-hand calcium binding domain 1
27	19659	0.0038081	-2.75	CSAG1	Chondrosarcoma associated gene 1
28	34931	0.0004351	-2.77	BCO2	Beta-carotene oxygenase 2
29	1588	0.0063379	-2.77	FAM59A	Family with sequence similarity 59
30	17838	0.0014638	-2.8	EVI1	Ecotropic viral integration site 1
31	2613	0.0092401	-2.8	GPR115	G protein-coupled receptor 115
32	39761	0.0012229	-2.86	P2RY1	Purinergic receptor P2Y
33	17290	0.0077798	-2.87	MCOLN3	Mucolipin 3
34	13247	0.0094448	-2.87	ANKRD29	Ankyrin repeat domain 29
35	14134	0.0037823	-2.88	MBNL3	Muscleblind-like 3 (Drosophila)
36	8193	0.0032315	-2.94	CNTNAP3	Similar to cell recognition molecule CASPR3
37	25913	0.0036957	-2.94	SEPP1	Selenoprotein P
38	13477	0.0069691	-2.94	KIAA1543	Kiaa1543
39	32907	0.0032092	-2.98	SMOC1	SPARC related modular calcium binding 1
40	43398	0.0041367	-2.99	GPRC5A	G protein-coupled receptor
41	23837	0.0026947	-3	MATN2	Matrilin 2
42	21423	0.0074074	-3	NRTN	Neurturin
43	25848	0.0009321	-3.01	B3GAT1	Beta-1
44	29722	0.0033029	-3.04	FAM55A	Family with sequence similarity 55
45	30076	0.0044174	-3.06	EPHA4	EPH receptor A4
46	12780	0.0010088	-3.07	IL18R1	Interleukin 18 receptor 1
47	38171	0.003897	-3.08	TFPI2	Tissue factor pathway inhibitor 2
48	33418	0.0034212	-3.12	CDH7	Cadherin 7
49	18011	0.0017524	-3.13	KLHL13	Kelch-like 13 (Drosophila)
50	12172	0.003645	-3.2	TTC39A	Tetratricopeptide repeat domain 39A
51	24279	0.0040675	-3.22	FLRT3	Fibronectin leucine rich transmembrane protein 3
52	28237	0.009053	-3.22	AKR1B10	Aldo-keto reductase family 1
53	17797	0.004425	-3.24	CXADR	Coxsackie virus and adenovirus receptor

54	13845	0.0088194	-3.29	AKR1C1	Aldo-keto reductase family 1
**Serial No.**	**ID**	**P Value**	**logFC**	**Gene Symbol**	**Gene title**
55	35486	0.0092782	-3.36	CPA6	Carboxypeptidase A6
56	3056	0.002141	-3.4	BTBD11	BTB (POZ) domain containing 11
57	21599	0.0084329	-3.46	CA9	Carbonic anhydrase IX
58	541	0.0003209	-3.47	PPP4R4	Protein phosphatase 4
59	32911	0.0061635	-3.48	RXFP1	Relaxin/insulin-like family peptide receptor 1
60	11872	0.0036382	-3.58	TMEM156	Transmembrane protein 156
61	39539	0.0025192	-3.59	SLC2A12	Solute carrier family 2 (facilitated glucose transporter)
62	32084	0.0000607	-3.65	C10orf82	Chromosome 10 open reading frame 82
63	22196	0.0017793	-3.66	C10orf116	Chromosome 10 open reading frame 116
64	33255	0.0096004	-3.85	AQP3	Aquaporin 3 (Gill blood group)
65	35009	0.0019334	-4.21	SLC6A15	Solute carrier family 6 (neutral amino acid transporter)
66	44018	0.000694	-4.31	PPARG	Peroxisome proliferator-activated receptor gamma
67	21986	0.0032162	-4.33	LPAR3	Lysophosphatidic acid receptor 3
68	21825	0.0074144	-4.37	VSIG2	V-set and immunoglobulin domain containing 2
69	39184	0.0024592	-4.47	COL4A6	Collagen
70	5959	0.0003276	-4.63	ANXA10	Annexin A10
71	34761	0.0011939	-4.66	RAB38	Rab38
72	10792	0.0001869	-4.74	HHIPL2	HHIP-like 2
73	40043	0.0001113	-4.75	DHRS3	Dehydrogenase/reductase (SDR family) member 3
74	19417	0.0001845	-4.93	SHANK2	SH3 and multiple ankyrin repeat domains 2
75	33803	0.0000177	-5.12	SNCA	Synuclein
76	1148	0.0000345	-5.44	RARRES1	Retinoic acid receptor responder (tazarotene induced) 1
