# Supplementary material for: Integrative Systems Biology Approaches to Identify Potential Biomarkers and Pathways of Cervical Cancer
Source: J Pers Med. 2021 Apr 30;11(5):363. doi: 10.3390/jpm11050363 (PMC8147030; doi:10.3390/jpm11050363)
Supplement: Supplementary file 1 [file jpm-11-00363-s001.zip › Table S3, S4, S5 & S6.pdf]

Table S3: The functional analyses of up-regulated genes to identify the top 10 GO terms.

Category	Term	Gene Count	Overlapped Gene	P Value
GO_BP	Immune response	23	CIITA, GPR183, CRIP1, CCL3, HLA-DRB1, CMKLR1, CCR1, HLA-DRB3, IL19, IL32, HLA-DQA2, C1QC, IL10, CCL18, SLC11A1, LILRB2, CCL23, RGS1, CCR5, FCGR1B, FCGR3A, HLA-DRA, LCP2	3.17E-11
GO_BP	Inflammatory response	20	HAVCR2, CIITA, CCL3, CCR1, IL19, LYZ, ITGB2, FPR3, IL10, CCL18, SLC11A1, CYBB, NLRC4, CCL23, CCR5, CXCR4, STAB1, CCL3L3, PLA2G4C, CSF1R	1.56E-09
GO_CC	Plasma membrane	69	KCNH1, MSR1, PLXNA4, CD53, DYSF, SLC1A3, CXCR4, FCGR3A, ANGPT2, PARVG, LAIR1, FMNL3, TMEM204, CMKLR1, PTPRRC, HLA-DQA2, CD163, CD84, LILRB2, CRHR2, CD163L1, CCR5, ARRB1, CD33, TREM2, SH3GL2, LCP1, GAP43, HLA-DRA, GPR183, HLA-DRB1, FGR, HLA-DRB3, CCR1, CYTH4, ITGA11, IFI30, OXTR, PCDHB14, ITGB2, FPR3, CDH4, EPHB1, KCNMB1, KCNMB2, SLC11A1, SORBS1, FAT3, TEK, FCGR1B, FCER1G, APBA2, SUCNR1, SELPLG, TYROBP, CSF1R, CARD9, GNAO1, CPPED1, PODXL, LSP1, CYBB, RGS1, STAB1, CD300A, RGS5, PECAM1, ECSCR, GFRA1	6.52E-08
GO_CC	Integral component of plasma membrane	34	KCNH1, GPR183, MSR1, PLXNA4, HLA-DRB1, HLA-DRB3, CCR1, OXTR, FPR3, CD53, CDH4, EPHB1, KCNMB2, SLC11A1, TEK, FCER1G, PCDHA11, SELPLG, TYROBP, CSF1R, PTPRB, CMKLR1, PODXL, HLA-DQA2, DCSTAMP, CD163, CD84, CRHR2, LILRB2, CYBB, CCR5, STAB1, CD33, HLA-DRA	3.88E-07
GO_BP	Positive regulation of ERK1 and ERK2 cascade	12	HAVCR2, GPR183, CCL3, CCL23, ARRB1, CCR1, CCL3L3, TEK, FGF1, TREM2, CCL18, CSF1R	5.58E-07
GO_BP	Leukocyte migration	10	CD84, PODXL, PECAM1, TEK, FCER1G, FPR3, ITGB2, ANGPT2, SELPLG, MMP1	1.64E-06
GO_BP	Chemokine-mediated signaling pathway	8	CCL3, CCL23, CCR5, CMKLR1, CXCR4, CCR1, CCL3L3, CCL18	3.55E-06
GO_BP	Cell adhesion	17	PARVG, CCR1, PODXL, ITGA11, IL32, ITGB2, AJAP1, CDH4, SORBS1, SORBS2, STAB1, CD300A, CD33, PECAM1, PCDHA11, LOXL2, SELPLG	4.24E-06
GO_MF	Actin binding	13	PARVG, FMNL3, NCALD, DAAM2, TPM1, LSP1, SORBS1, TAGLN, CCR5, GMFG, CXCR4, CNN1, LCP1	7.71E-06
GO_BP	Chemotaxis	9	LSP1, CCL3, CCL23, CCR5, CMKLR1, CXCR4, CCR1, ECSCR, CCL18	1.51E-05

Table S4: The functional analyses of down-regulated genes to identify the top 10 GO terms.

Category	Term	Gene Count	Overlapped Gene	P Value
GO_CC	Basolateral plasma membrane	14	PALM, SLC8A2, SLC7A8, CFTR, ATP12A, CXADR, AQP3, CD1D, CA9, NDRG4, P2RY1, MAP7, SLC40A1, PIANP	9.60E-06
GO_CC	Extracellular region	50	NRTN, GDF7, EFNA1, NPNT, IL18, FST, BTC, IGFBP6, SNCA, FGF10, CXADR, SERPINA3, CNTNAP3, PRSS37, KLK13, HHIPL2, COLEC10, DLL1, WFDC12, MELTF, CNTN4, WFDC2, CCK, CRHR1-IT1, C6, CLU, KITLG, FAM132A, CXCL6, MDK, EPHB2, LIF, PRRG4, PRSS2, PRSS3, PTH, TNFRSF18, DEFB1, ANGPTL4, KLK6, DEFB103B, MUC20, HGF, COL4A6, B3GAT1, LAMA3, PRSS27, WIF1, IGFBP2, NXPE1	2.09E-05
GO_CC	Extracellular space	44	SPINK13, CCK, LYPD3, SPOCK3, MSMB, GDF7, IL18, SNCA, CLU, IGFBP6, BTC, SORL1, FAM20C, KITLG, FGF10, FAM132A, CXCL6, GPRC5B, CXADR, CPZ, ALDH3A1, LIF, PRSS2, PTH, PRSS3, SERPINA3, SEMA3B, DEFB1, KLK13, ANGPTL4, FLRT3, KLK6, CPA6, LGALS4, DEFB103B, KLK5, PIGR, HGF, TNFSF9, MUC4, MELTF, CMTM8, IGFBP2, WFDC2	2.32E-05
GO_BP	Retinal metabolic process	5	AKR1C3, RDH10, ALDH1A3, AKR1C1, BCO2	3.47E-05
GO_MF	Receptor binding	17	FRK, NRTN, EFNA1, IGFBP6, LRRC4B, CXADR, TNFSF9, EPHB2, LIF, RND1, LAMA3, ITGB8, PTK6, C1QL1, MAP7, IGFBP2, SLC27A2	2.94E-04
GO_MF	Gamma-glutamyltransferase activity	4	GGT5, GGT3P, GGTLC2, GGTLC1	5.02E-04
GO_MF	Indanol dehydrogenase activity	3	AKR1C3, AKR1B10, AKR1C1	8.19E-04

Category	Term	Gene Count	Overlapped Gene	P Value
GO_BP	Activation of MAPK activity	8	DUSP5, ADORA2B, EFNA1, MUC20, LPAR3, FGF10, MAPK10, HGF	0.002184644
GO_MF	Retinol dehydrogenase activity	4	AKR1C3, RDH10, DHRS3, ADH1A	0.003093998
GO_BP	Leukotriene biosynthetic process	4	GGT5, GGT3P, GGTLC2, GGTLC1	0.004323082

Table S5: The gene lists of the significantly enriched KEGG pathways

Term	Gene Count	Overlapped Gene	P Value
Rheumatoid arthritis	10	CCL3, HLA-DRB1, HLA-DRB3, CCL3L3, TEK, ITGB2, ATP6V0D2, HLA-DQA2, MMP1, HLA-DRA	7.45E-07
Asthma	6	HLA-DRB1, HLA-DRB3, FCER1G, HLA-DQA2, IL10, HLA-DRA	2.26E-05
Chemokine signaling pathway	11	CCL3, CCL23, PLCB4, CCR5, FGR, CXCR4, NCF1, ARRB1, CCR1, CCL3L3, CCL18	5.82E-05
Osteoclast differentiation	9	LILRB2, CYBB, LILRB5, NCF1, FCGR3A, TREM2, CSF1R, TYROBP, LCP2	1.40E-04
Intestinal immune network for IgA production	6	HLA-DRB1, CXCR4, HLA-DRB3, HLA-DQA2, IL10, HLA-DRA	2.08E-04
Phagosome	9	MSR1, HLA-DRB1, NCF1, HLA-DRB3, ITGB2, FCGR3A, ATP6V0D2, HLA-DQA2, HLA-DRA	3.55E-04
Pathways in cancer	15	PTGER3, PTGS2, PPARG, SMAD3, KITLG, FGF10, LPAR3, LPAR2, HGF, MAPK10, MECOM, STAT3, COL4A6, LAMA3, LPAR5	0.003773286
Rap1 signaling pathway	10	ADORA2B, LPAR5, EFNA1, P2RY1, SIPA1L2, KITLG, LPAR3, FGF10, LPAR2, HGF	0.006259875
Metabolism of xenobiotics by cytochrome P450	6	UGT1A6, ALDH1A3, ADH1A, AKR1C1, DHDH, ALDH3A1	0.006407175
Chemical carcinogenesis	6	UGT1A6, CYP3A7, PTGS2, ALDH1A3, ADH1A, ALDH3A1	0.008868448

Term	Gene Count	Overlapped Gene	P Value
Retinol metabolism	5	UGT1A6, RDH10, DHRS3, CYP3A7, ADH1A	0.018866178
Drug metabolism - cytochrome P450	5	UGT1A6, ALDH1A3, FMO3, ADH1A, ALDH3A1	0.023042317
Cell adhesion molecules (CAMs)	7	LRRC4, F11R, OCLN, ITGB8, LRRC4B, CNTNAP2, CLDN23	0.0258561
Neuroactive ligand-receptor interaction	10	MCHR1, GABRE, PTGER3, RXFP1, ADORA2B, PRSS2, P2RY1, PRSS3, LPAR3, LPAR2	0.03270952
PI3K-Akt signaling pathway	11	LAMA3, LPAR5, ITGB8, EFNA1, KITLG, LPAR3, FGF10, LPAR2, HGF, BCL2L11, COL4A6	0.0485844
PPAR signaling pathway	4	PPARG, SLC27A6, SLC27A2, ANGPTL4	0.09149798
