# Supplementary material for: Integrative Systems Biology Approaches to Identify Potential Biomarkers and Pathways of Cervical Cancer
Source: J Pers Med. 2021 Apr 30;11(5):363. doi: 10.3390/jpm11050363 (PMC8147030; doi:10.3390/jpm11050363)
Supplement: Supplementary file 1 [file Figure S1.pdf › Figure S1.pdf]

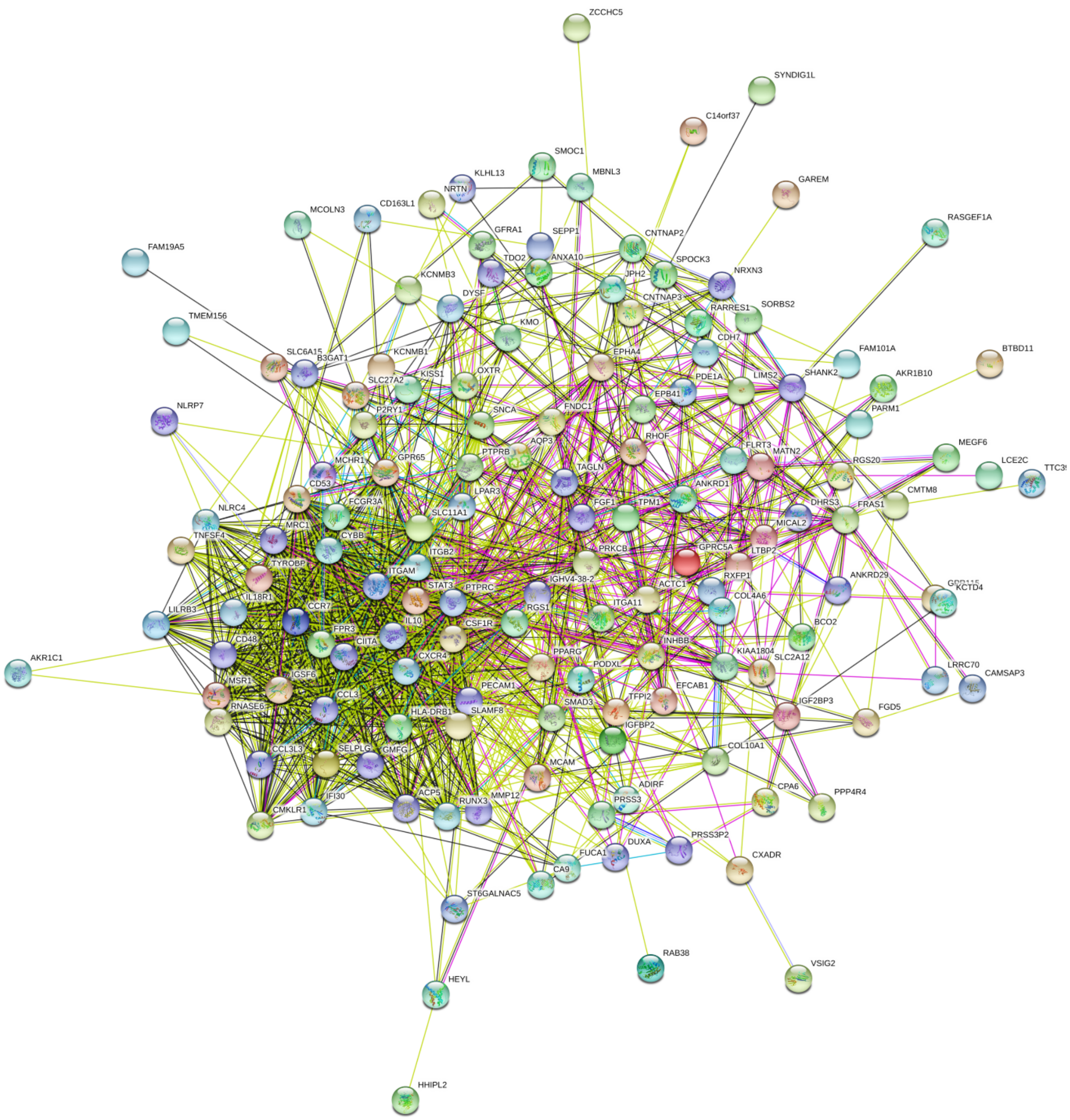

Figure S1: All differentially expressed genes in PPI network as a merged illustration. This PPI network had 1206 edges for 151 nodes.
